# Invariant NKT Cells From Donor Lymphocyte Infusions (DLI-iNKTs) Promote *ex vivo* Lysis of Leukemic Blasts in a CD1d-Dependent Manner

**DOI:** 10.3389/fimmu.2019.01542

**Published:** 2019-07-09

**Authors:** Simona Jahnke, Hannes Schmid, Kathy-Ann Secker, Jakob Einhaus, Silke Duerr-Stoerzer, Hildegard Keppeler, Irmtraud Schober-Melms, Rebecca Baur, Michael Schumm, Rupert Handgretinger, Wolfgang Bethge, Lothar Kanz, Corina Schneidawind, Dominik Schneidawind

**Affiliations:** ^1^Department of Hematology and Oncology, University Hospital Tuebingen, Tuebingen, Germany; ^2^Department of Hematology and Oncology, University of Erlangen-Nuremberg, Erlangen, Germany; ^3^Department of Hematology and Oncology, Children's University Hospital, Tuebingen, Germany

**Keywords:** DLI, GVHD, GVL, hematopoietic cell transplantation, iNKT cells, adoptive immunotherapy

## Abstract

Allogeneic hematopoietic cell transplantation (allo-HCT) is a curative treatment option for hematologic malignancies but relapse remains the most common cause of death. Infusion of donor lymphocytes (DLIs) can induce remission and prolong survival by exerting graft-vs.-leukemia (GVL) effects. However, sufficient tumor control cannot be established in all patients and occurrence of graft-vs.-host disease (GVHD) prevents further dose escalation. Previous data indicate that invariant natural killer T (iNKT) cells promote anti-tumor immunity without exacerbating GVHD. In the present study we investigated lysis of leukemic blasts through iNKT cells from donor-derived lymphocytes for leukemia control and found that iNKT cells constitute about 0.12% of cryopreserved donor T cells. Therefore, we established a 2-week cell culture protocol allowing for a robust expansion of iNKT cells from cryopreserved DLIs (DLI-iNKTs) that can be used for further preclinical and clinical applications. Such DLI-iNKTs efficiently lysed leukemia cell lines and primary patient AML blasts *ex vivo* in a dose- and CD1d-dependent manner. Furthermore, expression of CD1d on target cells was required to release proinflammatory cytokines and proapoptotic effector molecules. Our results suggest that iNKT cells from donor-derived lymphocytes are involved in anti-tumor immunity after allo-HCT and therefore may reduce the risk of relapse and improve progression-free and overall survival.

## Introduction

Allogeneic hematopoietic cell transplantation (allo-HCT) is a curative treatment option for many advanced or high-risk hematologic malignancies like acute myeloid leukemia (AML). Overall survival of such patients has improved over the last decades, but relapse remains the most common cause of death after allo-HCT. Allogeneic donor lymphocytes play an important role in disease control after allo-HCT as they may unfold potent graft-vs.-leukemia (GVL) effects. Infusions of donor lymphocytes (DLIs) from the original hematopoietic stem cell donor after allo-HCT were found to further strengthen GVL effects. This was first observed by Hans-Jochem Kolb and co-workers in chronic myeloid leukemia (CML) patients in the 1990s (1). Today, DLIs are of clinical use in case of mixed donor chimerism, minimal residual disease (MRD), or relapse. However, the administration of donor lymphocytes is complicated by the high risk of inducing acute or chronic graft-vs.-host disease (GVHD).

Invariant natural killer T (iNKT) cells are characterized by a semi-invariant T-cell receptor (TCRα Vα24-Jα18) with high affinity to glycolipids such as α-galactosylceramide (α-GalCer). Upon stimulation of their TCR, iNKT cells release high amounts of immunoregulatory cytokines such as IFN-γ, TNF-α, and IL-4. This enables iNKT cells to rapidly interact with lymphoid (B, T, and NK cells) and myeloid cells (monocytes, granulocytes), therefore, representing key players in the immuno-regulatory network (2–4). In addition, iNKT cells may induce cell death by producing granzyme B and perforin (5, 6), through Fas/FasL interactions (7–12), and TNF-α-mediated cytotoxic pathways (13). It was recently shown that high iNKT-cell numbers in peripheral blood stem cell grafts are associated with a reduced incidence of GVHD (14, 15) and an improved GVHD-free and progression-free survival (16). Therefore, we analyzed the cellular components of DLIs and investigated whether culture-expanded iNKT cells from DLIs (DLI-iNKTs) could be a way of enhancing anti-leukemia cytotoxicity and thus, help control relapse after allo-HCT.

## Materials and Methods

### Research Subjects

Cryopreserved human donor lymphocytes were obtained from the joint stem cell laboratories of the Department of Medicine II and Children's University Hospital Tübingen. These donor lymphocytes were collected by leukapheresis from 2012 to 2019. Primary leukemia cells (purity ≥ 90%) were cryopreserved from untreated patients after informed consent was obtained. The study was approved by our institutional review board to be in accordance with the ethical standards and with the Helsinki Declaration of 1975, as revised in 2013 (IRB approvals 137/2017BO2 and 887/2017BO2).

### Flow Cytometry Analysis

PBS57-loaded and unloaded human CD1d tetramers were obtained from the National Institutes of Health Tetramer Core Facility (Atlanta, Georgia, USA). DLIs and iNKT-cell cultures were analyzed by staining with the following antibodies purchased from BioLegend (San Diego, California, USA), BD Biosciences (Franklin Lakes, New Jersey, USA), or eBioscience (Waltham, Massachusetts, USA): CD3 (OKT3, PerCP/Cy5.5), CD4 (RPA-T4, BV785 or BV421), CD8a (HIT8a, AF700 or FITC), CD45 (HI30, BV650), CD19 (SJ25C1, APC-Cy7). Anti-human CD1d APC (Clone 51.1, BioLegend) was used to determine CD1d expression on leukemia cell lines and primary leukemia cells. Fixable Viability Dye eFluor506 from eBioscience and 7-aminoactinomycin (7-AAD, BD Biosciences) were used to exclude dead cells. Anti-human CD107a APC (H4A3, Biolegend) was used for CD107a degranulation assays. Data were acquired on an LSR Fortessa flow cytometer (BD Biosciences) and analysis was performed with FlowJo 10.2 (Tree Star, La Jolla, California, USA).

### iNKT-Cell Expansion

iNKT cells from human DLIs were expanded in iNKT-cell culture medium consisting of RPMI 1640 GlutaMAX™ Medium (ThermoFisher Scientific, Waltham, Massachusetts, USA), 10% FBS (fetal bovine serum, Biochrom, Berlin, Germany), 100 IU/ml penicillin-streptomycin (Lonza, Basel, Switzerland), 5.5 μM 2-mercaptoethanol (Roth, Karlsruhe, Germany), 0.1 mM non-essential amino acids (NEAA, Gibco, New York, New York, USA), 10 mM HEPES (Gibco) and 1 mM sodium pyruvate (Gibco). Donor lymphocytes were co-incubated with 100 ng/ml α-GalCer (Sigma-Aldrich, St. Louis, Missouri, USA) and 100 IU/ml recombinant human interleukin 2 (rhIL-2, Novartis, Basel, Switzerland). At day 7, rhIL-2 (100 IU/ml) and α-GalCer (100 ng/ml) was added to the culture and iNKT cells were re-stimulated with irradiated (30 Gy, cesium-137 irradiator Gammacell 1000, Atomic Energy of Canada Limited, Chalk River, Ontario, Canada) and glycolipid-pulsed autologous or allogeneic peripheral blood mononuclear cells (PBMCs) for another 7 days. Thereafter, iNKT-cell expansion was completed. At days 7 and 14, viability and percentage of DLI-iNKTs were measured by flow cytometry.

### Magnetic-Activated Cell Sorting (MACS)

For purification of DLI-iNKTs, staining with PBS57-CD1d Tetramer PE was performed. Anti-PE-Microbeads UltraPure (Miltenyi Biotec, Bergisch Gladbach, Germany) were used to enrich DLI-iNKTs via QuadroMACS™ Separator (Miltenyi Biotec) and LS Columns (Miltenyi Biotec) according to the manufacturer's instructions.

### Tumor Cell Lysis Assay

DLI-iNKTs were co-incubated with leukemia cell lines or primary patient leukemia cells at increasing effector- to target-cell ratios. The following tumor cell lines were used as target cells: Jurkat (Clone E6-1, ATCC, Manassas, Virginia, USA), K562 (CCL-243, ATCC, Manassas, Virginia, USA), THP-1 (TIB-202, ATCC, Manassas, Virginia, USA). Co-culture was performed in iNKT-cell culture medium with and without 100 ng/ml α-GalCer. After 16 h, cell lysis was measured by flow cytometry (LSR Fortessa, BD Biosciences) using 7-aminoactinomycin (7-AAD, BD Biosciences) and iNKT cells were excluded by staining with PBS57-loaded human CD1d tetramer. Specific lysis was calculated by the following formula: percentage of specific lysis = [1—(target cell viability with effector cells/target cell viability without effector cells)] ×100. For blocking experiments, purified anti-human CD1d (51.1, BioLegend) and the respective isotype control antibody were used at 10 μg/ml.

### CD107a Assay

DLI-iNKTs were co-incubated with target cells at a 2.5:1 ratio in presence of anti-human CD107a APC (H4A3, Biolegend) and protein transport inhibitor cocktail (Brefeldin A und Monensin, 500X, eBioscience). For blocking experiments, anti-CD1d or the respective isotype control were added. After 16 h, additional staining with PBS57-CD1d Tetramer and 7-AAD was performed and cells were measured using an LSR Fortessa (BD Bioscience).

### Cytokine Analysis

Supernatants from tumor cell lysis experiments were collected after 16 h and stored at −20°C. A multiplex assay (LEGENDplex™ Human CD8/NK Panel (13-plex), BioLegend) was used according to the manufacturer's instructions. LEGENDplex™ Software from BioLegend was used for analysis of acquired data.

### Statistical Analysis

Flow cytometry data were analyzed by FlowJo V10 (Treestar). Data were further analyzed with Prism 7.01 (GraphPad Software, La Jolla, CA, USA). Experiments were performed in duplicates and repeated independently at least three times. Student's *t*-test and one-way ANOVA were used for statistical analysis and *p* < 0.05 was considered statistically significant.

## Results

### DLIs Contain a Small but Distinct Fraction of Mostly CD4^−^/CD8^−^ iNKT Cells

**Figure 1:**
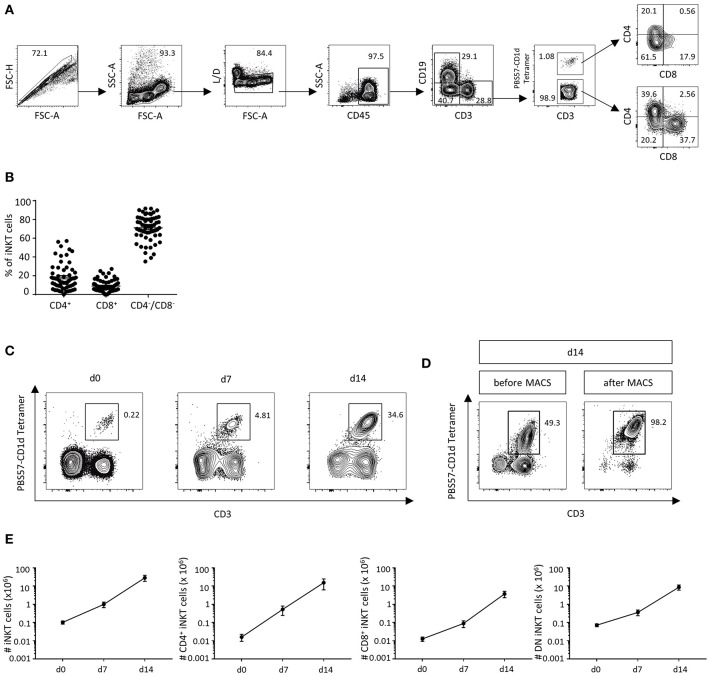
DLIs contain low numbers of iNKT cells that can be expanded *ex vivo*. **(A)** Gating strategy to identify CD3^+^PBS57-CD1d Tetramer^+^ iNKT cells and CD4^+^CD8^−^, CD4^−^CD8^+^ and CD4^−^CD8^−^ iNKT-cell subsets in DLIs. **(B)** Percent of iNKT-cell subsets in DLIs prior to *ex vivo* expansion (*n* = 63). Bars represent standard error of the mean (SEM) **(C)**. Representative dot plots showing iNKT-cell expansion following a 2-week cell culture protocol using α-GalCer and rhIL-2 **(D)**. Representative dot plots illustrating further purification of DLI-iNKTs by magnetic-activated cell sorting (MACS) after 14 days of cell culture **(E)**. Absolute numbers of iNKT cells and iNKT-cell subsets at 0, 7, and 14 days of cell culture (*n* = 7). Bars represent SEM.

In order to analyze the amount of T cells and iNKT cells in human DLIs (*n* = 63) by flow cytometry, the gating strategy was applied as outlined in Figure 1A. CD3^+^ T cells represent 47.3% of living cells (SD ± 16.0%). A small but distinct fraction of iNKT cells was detected in human DLIs, constituting 0.12% of CD3^+^ T cells (SD ± 0.22%). We then analyzed iNKT-cell subtypes and found that most iNKT cells were CD4^−^CD8^−^ (71.1% of iNKT cells, SD ± 13.4%, Figure 1B). 18.4% were CD4^+^ iNKT cells (SD ± 14.1%) and 9.1% were CD8^+^ iNKT cells (±6.3%). Administration of granulocyte colony-stimulating factor (G-CSF, Lenograstim, 2 × 5 μg/kg/d for 5 days) prior to collection of donor lymphocytes did not affect iNKT-cell numbers, subsets and function (Supplemental Figure 1).

### Glycolipid Stimulation for 2 Weeks Leads to Stable *ex vivo* Expansion of Human iNKT Cells From Cryopreserved DLIs

Due to the low cell numbers of iNKT cells in cryopreserved DLIs, we established a 2-week expansion protocol to obtain enough cells for further experiments and potential clinical applications. A 281-fold expansion (range 71.4–696.6) of iNKT cells with a purity of 24.7% was obtained after 2 weeks of cell culture using the glycolipid α-GalCer and rhIL-2 (Figure 1C). Subsequently, we further enriched culture-expanded DLI-iNKTs by magnetic-activated cell sorting (MACS, Figure 1D) and reached a purity of >95%. Absolute iNKT-cell counts from seven independent experiments are shown in Figure 1E. Moreover, we observed a preferential expansion of CD4^+^ iNKT cells (969-fold expansion compared to 297-fold expansion of CD8^+^ and 122-fold expansion of CD4^−^CD8^−^ iNKT cells, *n* = 7, Figure 1E).

### DLI-iNKTs Lyse Leukemia Cell Lines in a Dose-Dependent Manner and Upregulate the Degranulation Marker CD107a

**Figure 2:**
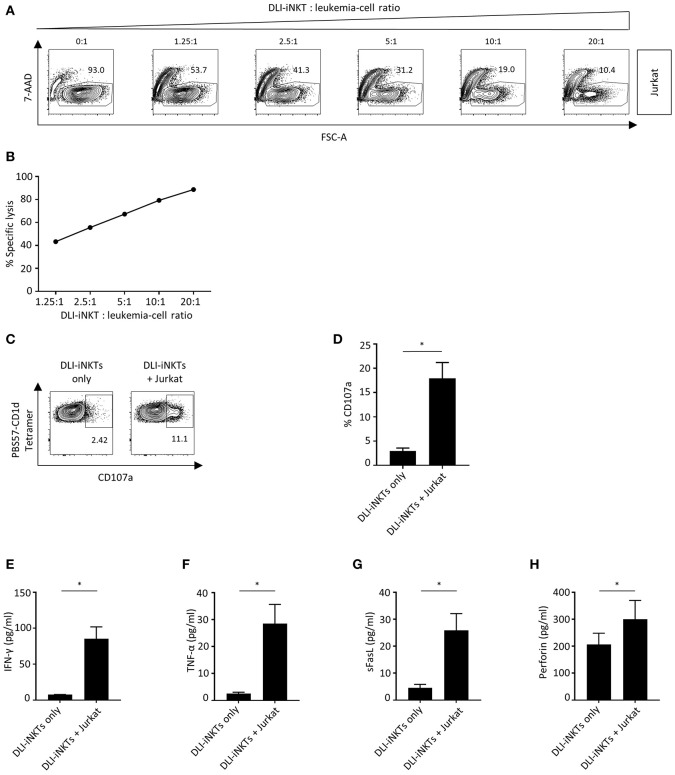
DLI-iNKTs lyse Jurkat cells in a dose-dependent manner. **(A)** Representative dot plots of DLI-iNKT-induced lysis of Jurkat cells in presence of α-GalCer. iNKT cells were excluded by gating on PBS57-CD1d Tetramer^−^ cells. **(B)** Specific lysis of Jurkat cells co-cultured with increasing numbers of DLI-iNKTs in presence of α-GalCer. Shown is one of seven representative experiments. **(C)** Representative dot plots and **(D)** pooled data illustrating CD107a expression on CD3^+^PBS57-CD1d Tetramer^+^ DLI-iNKTs after co-culture with Jurkat cells in presence of α-GalCer (*n* = 3). **(E)** IFN-γ, **(F)** TNF-α, **(G)** sFasL, and **(H)** perforin measured in supernatants after co-culture with Jurkat cells in presence of α-GalCer (*n* = 5). Bars represent SEM. ^*^p < 0.05.

Next, we were interested whether culture-expanded and purified DLI-iNKTs could exert anti-leukemia activity being crucial for disease control after allo-HCT. Therefore, DLI-iNKTs were co-incubated with Jurkat leukemia cells at increasing effector- to target-cell ratios. We observed a dose-dependent lysis of Jurkat cells (Figures 2A,B) that was more pronounced in presence of α-GalCer (Supplemental Figures 2A,B).

CD107a (LAMP-1) is a degranulation marker expressed on activated cytotoxic T cells (17–19) and NK cells (20) and has been shown to correlate with cytotoxicity (21). CD107a is also expressed on iNKT cells (22). We found a significantly higher expression of CD107a upon engagement with Jurkat cells compared to DLI-iNKTs alone indicating the release of cytotoxic effector molecules (Figures 2C,D). Supplemental Figures 2C,D demonstrate an increased upregulation of CD107a on DLI-iNKTs in presence of α-GalCer compared to without glycolipid. Moreover, upregulation of CD107a was most pronounced on the CD4-CD8- subset of DLI-iNKTs (Supplemental Figure 4).

The functional hallmark of iNKT cells is the instant release of immunoregulatory cytokines. Therefore, proinflammatory cytokines and proapoptotic effector molecules such as IFN-γ, TNF-α, and perforin were analyzed after co-culture of DLI-iNKTs and target cells. We observed a significantly increased production of IFN-γ (Figure 2E), TNF-α (Figure 2F), sFasL (Figure 2G), and perforin (Figure 2H) when DLI-iNKT cells were co-incubated with Jurkat cells compared to DLI-iNKT cells without target cells.

### DLI-iNKTs Lyse Leukemia Cells in a CD1d-Dependent Manner

**Figure 3:**
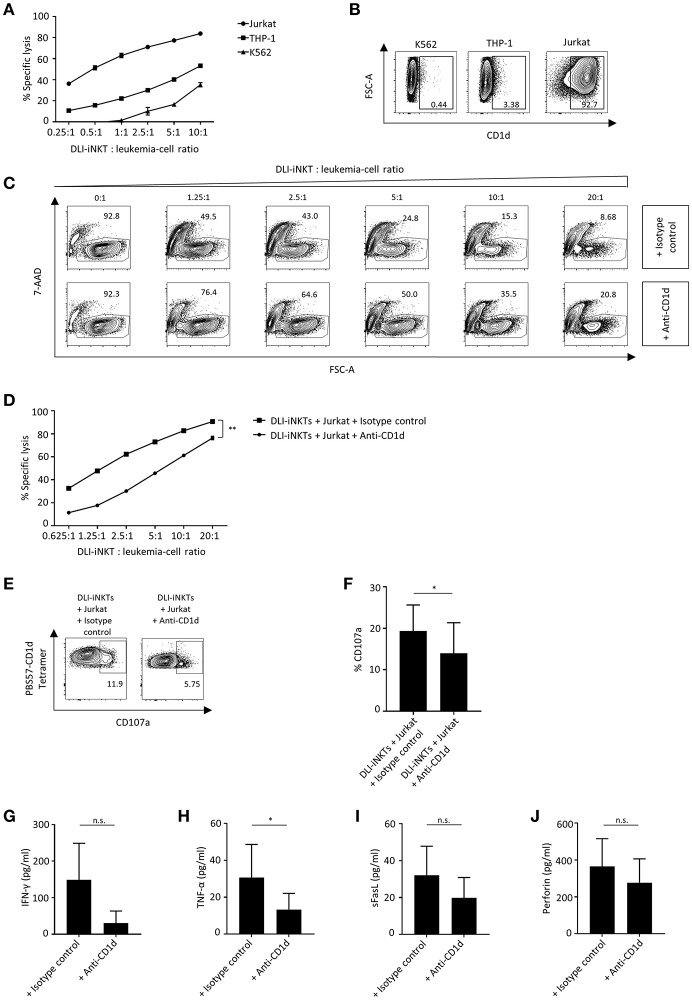
CD1d expression is required for efficient leukemia cell lysis through DLI-iNKTs. **(A)** Specific lysis of Jurkat cells, THP-1 and K562 co-cultured with increasing numbers of DLI-iNKTs in presence of α-GalCer (*n* = 2). **(B)** Representative dot plots illustrating CD1d expression on K562, THP-1, and Jurkat leukemia cell lines. **(C)** Representative dot plots and **(D)** specific lysis of Jurkat cells through DLI-iNKTs in presence of anti-CD1d and isotype control antibody together with α-GalCer (*n* = 3). iNKT cells were excluded by gating on PBS57-CD1d Tetramer^−^ cells. **(E)** Representative dot plots and **(F)** pooled data illustrating CD107a expression on CD3^+^PBS57-CD1d Tetramer^+^ DLI-iNKTs after co-culture with Jurkat cells and anti-CD1d or isotype control antibody in presence of α-GalCer (*n* = 3). **(G)** IFN-γ, **(H)** TNF-α, **(I)** sFasL, and **(J)** perforin measured in supernatants after co-culture with Jurkat cells and anti-CD1d or isotype control antibody in presence of α-GalCer (*n* = 5). Bars represent SEM. ^*^p < 0.05; ^**^p < 0.01.

We challenged various leukemia cell lines with DLI-iNKTs and found significant differences regarding the effectiveness of leukemia cell lysis: dose-dependent specific lysis of target cells was most efficient for Jurkat cells followed by THP-1 and K562 (Figure 3A). iNKT cells can be activated by TCR stimulation through presentation of glycolipids by the MHC class I-like molecule CD1d. Therefore, we were interested in determining the expression of CD1d on leukemia cell lines. We found that CD1d expression was highest on Jurkat cells, followed by THP-1 and almost no CD1d was expressed on K562 (Figure 3B).

Consequently, we assumed that CD1d expression on target cells is required to induce efficient leukemia cell lysis through DLI-iNKTs. Adding the CD1d-blocking antibody 51.1 resulted in a significantly decreased specific lysis of Jurkat cells (Figures 3C,D) and a significantly reduced expression of CD107a on DLI-iNKTs (Figures 3E,F) compared to the corresponding isotype control. We could also observe CD1d-dependent lysis in absence of α-GalCer (Supplemental Figure 3). In addition, we studied the release of proinflammatory cytokines and proapoptotic effector molecules while blocking CD1d on target cells: levels of IFN-γ (Figure 3G), TNF-α (Figure 3H), sFasL (Figure 3I), and perforin (Figure 3J) were decreased in presence of anti-CD1d compared to isotype control antibody. We conclude that DLI-iNKTs release cytotoxic effector molecules in a CD1d-dependent manner resulting in leukemia cell death.

### Primary Patient Leukemia Cells Are Lysed by Culture-Expanded DLI-iNKTs in a Dose- and CD1d-Dependent Manner

**Figure 4:**
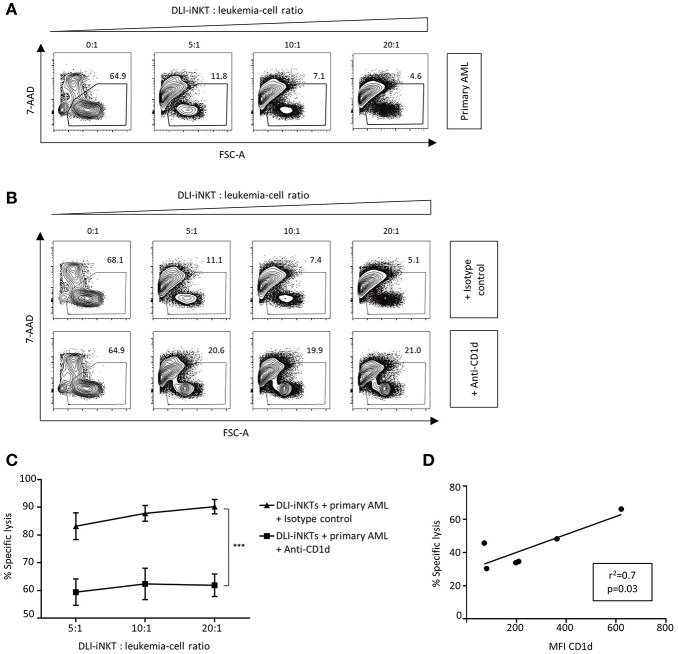
Patient AML blasts are lysed by DLI-iNKTs in a CD1d-dependent manner. **(A)** Representative dot plots illustrating dose-dependent lysis of primary patient AML blasts through culture-expanded DLI-iNKTs. **(B)** Representative dot plots and **(C)** specific lysis of primary patient AML blasts through DLI-iNKTs in presence of anti-CD1d and isotype control antibody together with α-GalCer (*n* = 3). iNKT cells were excluded by gating on PBS57-CD1d Tetramer^−^ cells. Bars represent SEM. **(D)** Correlation of specific lysis of primary patient AML blasts through DLI-iNKTs with the mean fluorescence intensity (MFI) of CD1d on respective leukemia cells (*n* = 6). ^***^p < 0.001.

Next, we investigated whether primary patient AML blasts were also susceptible to DLI-iNKT-induced cytotoxicity. When incubating different primary AML blasts from patients with DLI-iNKTs, we observed efficient and dose-dependent lysis of primary leukemia cells (Figure 4A). In line with our previous findings, blockade of CD1d significantly reduced leukemia cell lysis (Figures 4B,C). Comparable results were obtained without adding α-GalCer to the culture (Supplemental Figure 5). Accordingly, the expression level of CD1d on primary patient AML blasts significantly correlated with their specific lysis through DLI-iNKTs (*r*^2^ = 0.7, *p* = 0.03, Figure 4D). Therefore, higher expression of CD1d on leukemia cells could be an indicator of improved leukemia cell lysis and a prognostic factor for successful DLI-iNKT cytotherapy. Supplemental Figure 6 shows representative dot plots of CD1d expression and the immunophenotype of AML blasts used for this study.

## Discussion

DLIs are capable of inducing remission and converting mixed into complete donor chimerism after allo-HCT (23). Therefore, DLIs represent a widely accepted therapy for relapse and prevention of graft rejection. However, loss of immune tolerance and occurrence of GVHD often preclude administration of donor lymphocytes and further dose escalation. Previous clinical studies showed that higher iNKT-cell numbers in the graft or peripheral blood post-transplant were associated with a reduced incidence of GVHD (14, 24). De Lalla and co-workers investigated iNKT-cell reconstitution in pediatric haploidentical transplant patients and found that iNKT cells failed to reconstitute in individuals experiencing relapse (25). Moreover, increased iNKT-cell numbers in peripheral blood stem cell allografts correlated with an improved GVHD-free and progression-free survival indicating that iNKT cells induce immune tolerance while allowing for robust GVL effects (16). In addition, adoptively transferred iNKT cells prevent lethal GVHD without compromising T cell-mediated lysis of leukemia cells in murine models of allo-HCT (3, 26). In contrast, these iNKT-cell infusions exert potent anti-tumor immunity by themselves (3). Based on these observations we investigated the amount, expansion capacity, and functional properties of human iNKT cells from cryopreserved DLIs.

As iNKT cells are scarce in human peripheral blood and in freshly thawed DLIs, they first need to be expanded *in* or *ex vivo*. This could be done by intravenous administration of exogenous iNKT-cell agonists such as α-GalCer. However, intravenous infusion of α-GalCer may induce overshooting donor iNKT-cell activation that can result in iNKT-cell anergy and exhaustion (27), thus failing to show any clinical response. Like freshly thawed DLIs, transplant patients contain low iNKT-cell numbers due to the extensive pretreatment probably further limiting the effectiveness of glycolipid infusions. We therefore expand iNKT cells from cryopreserved DLIs following a 2-week protocol using α-GalCer and rhIL-2: although most iNKT cells were double negative before cell culture, we observed a preferential expansion of CD4^+^ cells. iNKT cells are a complex cell population with diverse subsets: CD4^−^ iNKT cells were shown to produce Th1 cytokines such as IFN-γ and TNF-α as well as double-negative iNKT cells that showed a Th1 profile (28). In contrast, CD4^+^ iNKT cells could produce both Th1 and Th2 cytokines (29). After allo-HCT, stimulation of CD4^+^ iNKT cells results in secretion of Th2-biased cytokines such as IL-4 and IL-13 that are both critical to restore immune tolerance (30, 31). However, CD4^+^ iNKT cells are also capable of lysing tumor cells releasing cytotoxic effector molecules (32, 33). Accordingly, we showed that DLI-iNKTs produce proinflammatory cytokines as well as perforin exerting potent anti-leukemia cytotoxicity that is dependent on the expression of CD1d on leukemia cells.

iNKT cells can be activated by direct interaction with tumor cells: CD1d^+^ tumor cells present endogenous glycolipids via CD1d which is then recognized by the T-cell receptor of iNKT cells leading to perforin/granzyme B or Fas/FasL-mediated cytotoxicity. CD1d is mostly expressed in hematopoietic cells (34, 35) and can be found on myelomonocytic and B-cell lineage malignancies (36). Accordingly, Spanoudakis et al. showed that myeloma progression is associated with decreased CD1d surface expression, linking CD1d with tumor survival in humans (37). Conversely, increasing the expression of antigen-presenting molecules like CD1d by gemcitabine and cyclophosphamide and combining chemotherapy with NKT-cell activation results in enhanced tumor control and survival (38). However, cross-presentation of tumor glycolipids by antigen-presenting cells (APCs) may also play an important role in iNKT-cell activation since some entities show low or no CD1d expression: presentation of glycolipids via CD1d on APCs stimulates iNKT cells to produce cytokines such as IFN-γ and IL-2 that subsequently activate NK cells and tumor-specific T cells (39). In the context of umbilical cord blood transplantation, Beziat et al. showed that iNKT cells efficiently lysed CD1d expressing blasts 6 months after transplant (40).

Importantly, our data indicate that lysis of leukemia cell lines and primary blasts is more efficient in presence of α-GalCer. Glycolipid-loaded tumor cells might be more visible to DLI-iNKTs while the respective ligand induces robust activation of effector cells. To enhance leukemia control after adoptive transfer of DLI-iNKTs, concomitant infusion of such glycolipids should be considered. Chen and colleagues have demonstrated the feasibility and safety of glycolipid infusions in the setting of allogeneic HCT (41).

We reported previously that G-CSF administration prior to donor lymphocyte apheresis results in an improved conversion to complete donor chimerism and a lower incidence of relapse or progression without increasing the risk of GVHD after infusion of donor lymphocytes. We also identified higher numbers of hematopoietic stem and progenitor cells, myeloid-derived suppressor cells and monocytes as independent risk factors for an improved overall survival (42). In the present study, 26 DLIs were derived from donors that were pretreated with G-CSF. However, G-CSF administration did not influence iNKT-cell numbers, subsets, expansion and lysis of leukemic blasts. Taken together, we assume that iNKT-cell numbers in conventional DLIs are too small to directly exert robust anti-leukemic effects. Instead, prior expansion and activation by glycolipids seems to be a reasonable approach to promote sustained GVL effects through iNKT cells themselves.

Therefore, in order to exploit potent GVL effects without exacerbating GVHD, manipulating the cellular composition of DLIs may be beneficial: by expanding iNKT cells *ex vivo*, they could be enriched in DLIs prior to infusion into patients. As GVHD represents a major dose-limiting toxicity and side effect of allo-HCT and DLIs, further dose escalation of donor lymphocytes is often impossible, therefore not allowing clinicians to completely harness the power of DLIs in leukemia control and prevention of relapse. iNKT cells represent a promising opportunity as they suppress GVHD without losing GVL effects (3, 16).

## Data Availability

The datasets generated for this study are available on request to the corresponding author.

## Ethics Statement

This study was carried out in accordance with the recommendations of the Helsinki Declaration of 1975, as revised in 2013 (IRB approvals 137/2017BO2 and 887/2017BO2) with written informed consent from all subjects. All subjects gave written informed consent in accordance with the Declaration of Helsinki. The protocol was approved by the institutional review board of the University Hospital Tuebingen, Germany.

## Author Contributions

SJ designed and performed research, analyzed data, and wrote the manuscript. HS, KA-S, JE, S-DS, HK, and RB performed experiments and analyzed data. IS-M performed the leukapheresis procedure and provided cell products. MS, RH, WB, LK, and CS helped interpreting data and assisted in preparing the manuscript. DS designed experiments, wrote the manuscript, and provided overall guidance. All authors edited the manuscript for content.

### Conflict of Interest Statement

The authors declare that the research was conducted in the absence of any commercial or financial relationships that could be construed as a potential conflict of interest. The reviewer GC and handling editor declared their shared affiliation.

## Supplementary Material

Supplemental Figure 1 Application of G-CSF before donor lymphocyte apheresis. Impact of prior G-CSF administration (*n* = 26) compared to steady state apheresis (*n* = 35) on **(A)** iNKT-cell numbers, **(B)** iNKT-cell subsets, **(C)** expansion of iNKT cells (*n* = 7), and **(D)** specific lysis of Jurkat cells by culture-expanded DLI-iNKTs (*n* = 8). Bars represent SEM.

Supplemental Figure 2 DLI-iNKTs lyse Jurkat cells in a dose-dependent manner. **(A)** Representative dot plots of DLI-iNKT-induced lysis of Jurkat cells without and with α-GalCer. iNKT cells were excluded by gating on PBS57-CD1d Tetramer^−^ cells **(B)**. Specific lysis of Jurkat cells co-cultured with increasing numbers of DLI-iNKTs without and with α-GalCer. Shown are pooled data of three representative experiments. **(C)** Representative dot plots and **(D)** pooled data illustrating CD107a expression on CD3^+^PBS57-CD1d Tetramer^+^ DLI-iNKTs after co-culture with Jurkat cells without and with α-GalCer (*n* = 3). Bars represent SEM. ^*^*p* < 0.05; ^***^*p* < 0.001.

Supplemental Figure 3 CD1d expression is required for efficient leukemia cell lysis through DLI-iNKTs. **(A)** Representative dot plots and **(B)** specific lysis of Jurkat cells through DLI-iNKTs in presence of anti-CD1d and isotype control antibody without and with α-GalCer (*n* = 3). iNKT cells were excluded by gating on PBS57-CD1d Tetramer^−^ cells. **(C)** Representative dot plots and **(D)** pooled data illustrating CD107a expression on CD3^+^PBS57-CD1d Tetramer^+^ DLI-iNKTs after co-culture with Jurkat cells and anti-CD1d or isotype control antibody without and with α-GalCer (*n* = 3). Bars represent SEM. ^***^*p* < 0.001.

Supplemental Figure 4 Expression of CD107a on DLI-iNKT subsets. Expression of CD107a on **(A)** CD4^+^CD8^−^ **(B)** CD4^−^CD8^+^ **(C)** CD4^−^CD8^−^ CD3^+^PBS57-CD1d Tetramer^+^ DLI-iNKTs after co-culture with Jurkat cells and anti-CD1d or isotype control antibody without and with α-GalCer. For each group *n* = 3. Bars represent SEM. ^*^*p* < 0.05.

Supplemental Figure 5 Patient AML blasts are lysed by DLI-iNKTs in a CD1d-dependent manner. **(A)** Representative dot plots and **(B)** specific lysis illustrating dose-dependent lysis of primary patient AML blasts through culture-expanded DLI-iNKTs in absence and in presence of α-GalCer (*n* = 3). **(C)** Representative dot plots and **(D)** specific lysis of primary patient AML blasts through DLI-iNKTs in presence of anti-CD1d and isotype control antibody together with and without α-GalCer (*n* = 3). iNKT cells were excluded by gating on PBS57-CD1d Tetramer^−^ cells. Bars represent SEM. ^*^*p* < 0.05.

Supplemental Figure 6 Phenotype of patient AML blasts. **(A)** Representative dot plots of CD1d staining. **(B)** Immunophenotype of patient AML blasts. 0, negative; 0/+, low; +, positive; n.a., data not available; pB, peripheral blood.
