# Supplementary figures and images for: Invariant NKT Cells From Donor Lymphocyte Infusions (DLI-iNKTs) Promote ex vivo Lysis of Leukemic Blasts in a CD1d-Dependent Manner
Source: Front Immunol. 2019 Jul 9;10:1542. doi: 10.3389/fimmu.2019.01542 (PMC6629940; doi:10.3389/fimmu.2019.01542)

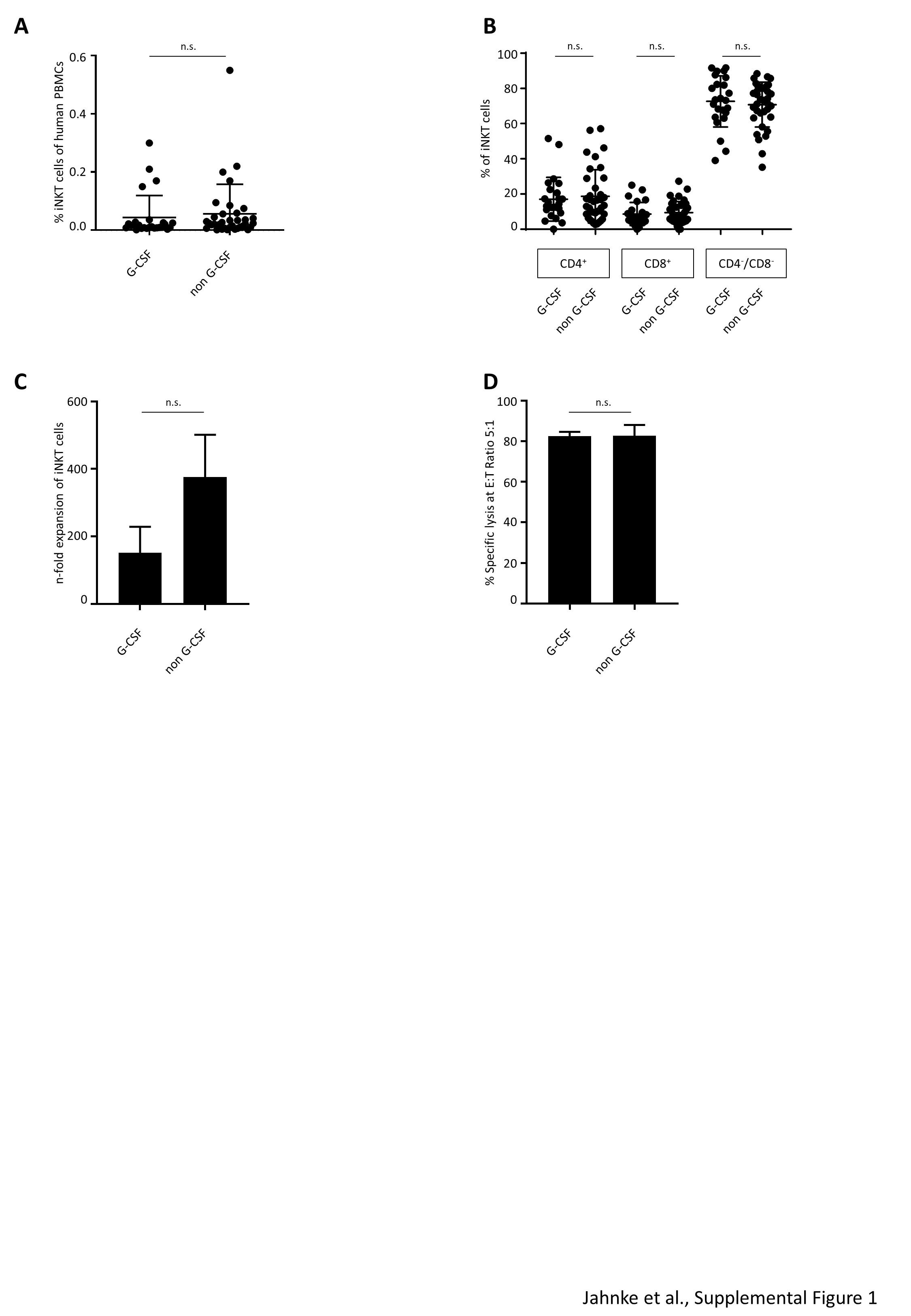

Supplement: Supplemental Figure 1 — Application of G-CSF before donor lymphocyte apheresis. Impact of prior G-CSF administration (n = 26) compared to steady state apheresis (n = 35) on (A) iNKT-cell numbers, (B) iNKT-cell subsets, (C) expansion of iNKT cells (n = 7), and (D) specific lysis of Jurkat cells by culture-expanded DLI-iNKTs (n = 8). Bars represent SEM. [file Image_1.JPEG]

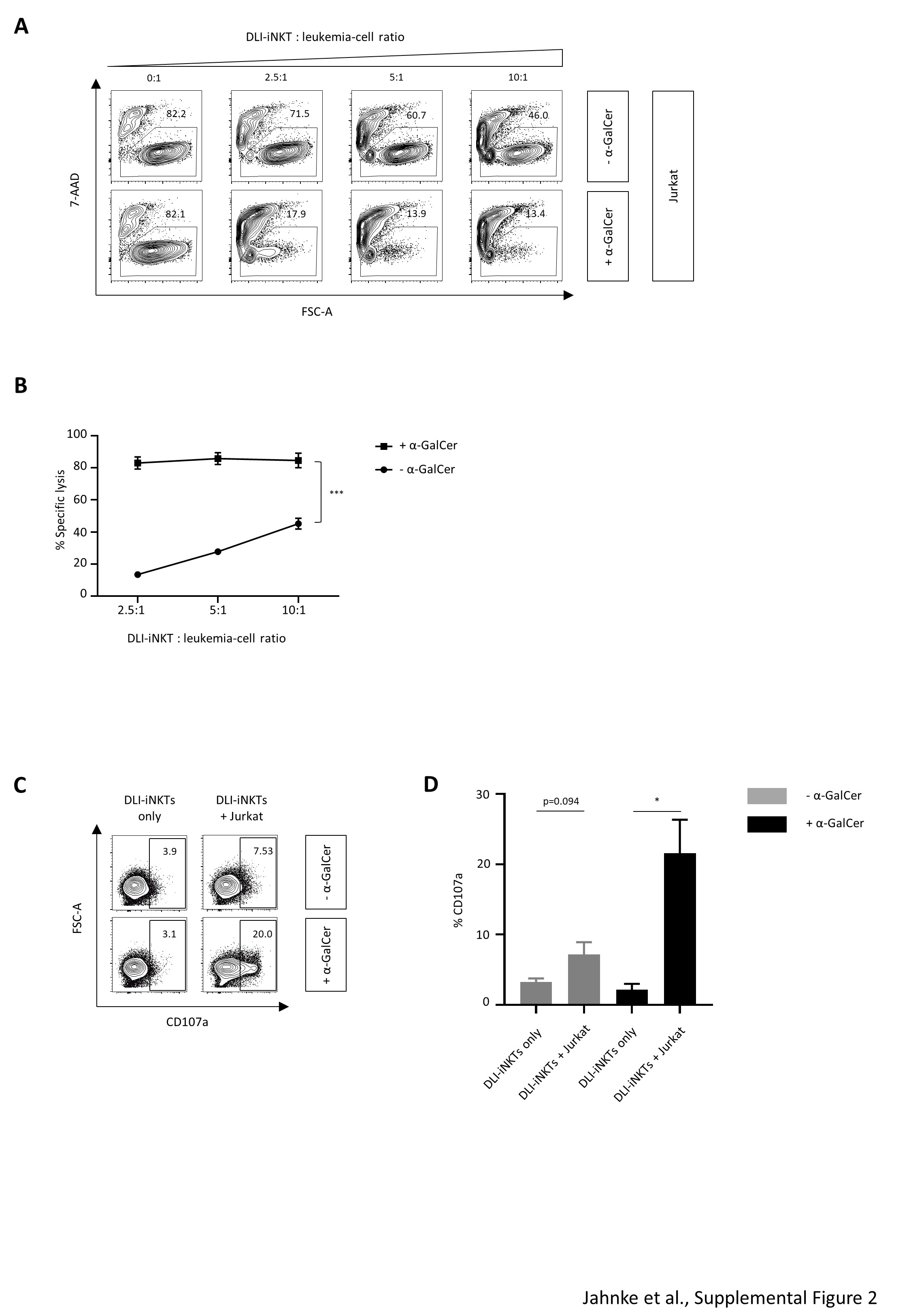

Supplement: Supplemental Figure 2 — DLI-iNKTs lyse Jurkat cells in a dose-dependent manner. (A) Representative dot plots of DLI-iNKT-induced lysis of Jurkat cells without and with α-GalCer. iNKT cells were excluded by gating on PBS57-CD1d Tetramer− cells (B). Specific lysis of Jurkat cells co-cultured with increasing numbers of DLI-iNKTs without and with α-GalCer. Shown are pooled data of three representative experiments. (C) Representative dot plots and (D) pooled data illustrating CD107a expression on CD3+PBS57-CD1d Tetramer+ DLI-iNKTs after co-culture with Jurkat cells without and with α-GalCer (n = 3). Bars represent SEM. *p < 0.05; ***p < 0.001. [file Image_2.JPEG]

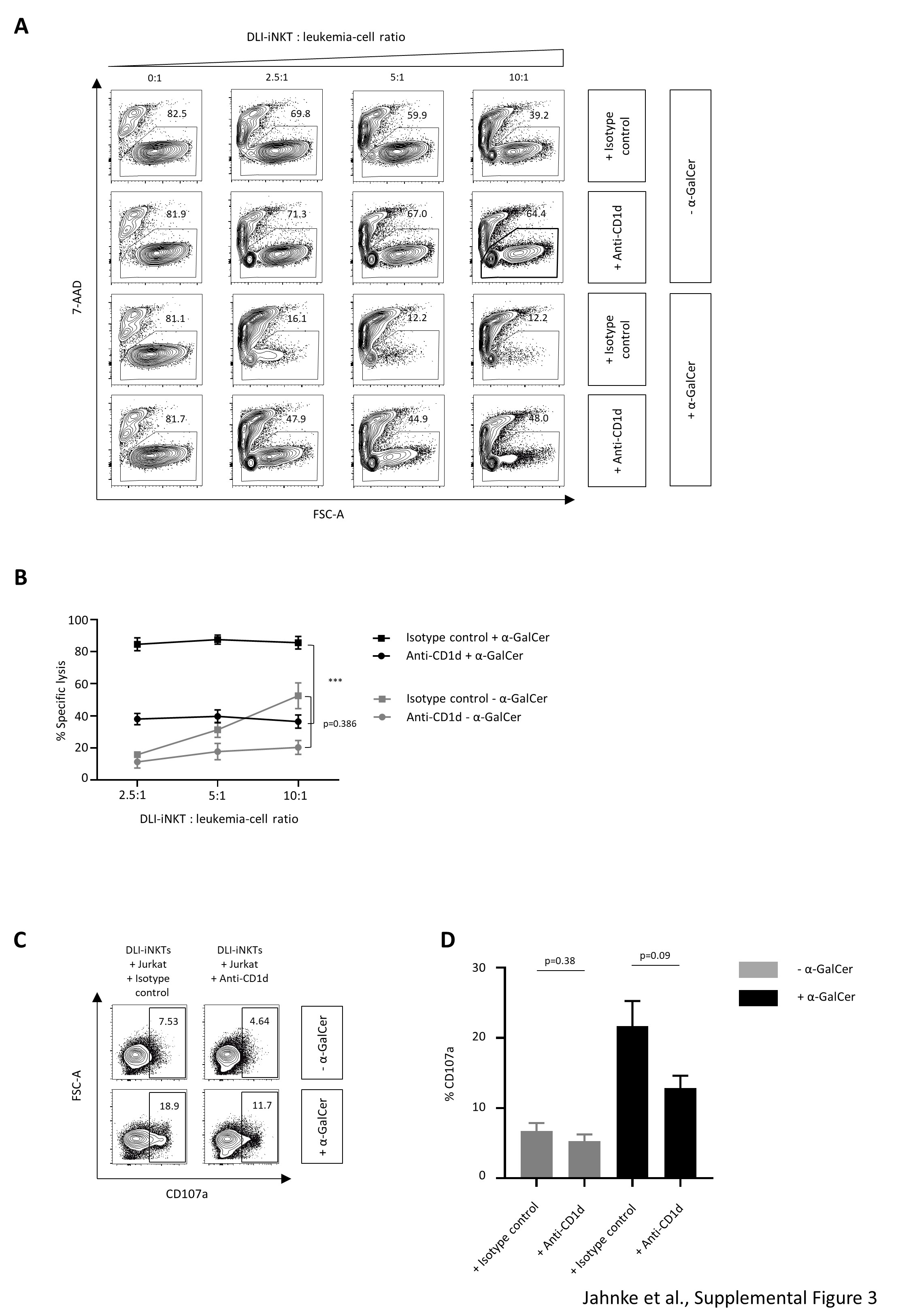

Supplement: Supplemental Figure 3 — CD1d expression is required for efficient leukemia cell lysis through DLI-iNKTs. (A) Representative dot plots and (B) specific lysis of Jurkat cells through DLI-iNKTs in presence of anti-CD1d and isotype control antibody without and with α-GalCer (n = 3). iNKT cells were excluded by gating on PBS57-CD1d Tetramer− cells. (C) Representative dot plots and (D) pooled data illustrating CD107a expression on CD3+PBS57-CD1d Tetramer+ DLI-iNKTs after co-culture with Jurkat cells and anti-CD1d or isotype control antibody without and with α-GalCer (n = 3). Bars represent SEM. ***p < 0.001. [file Image_3.JPEG]

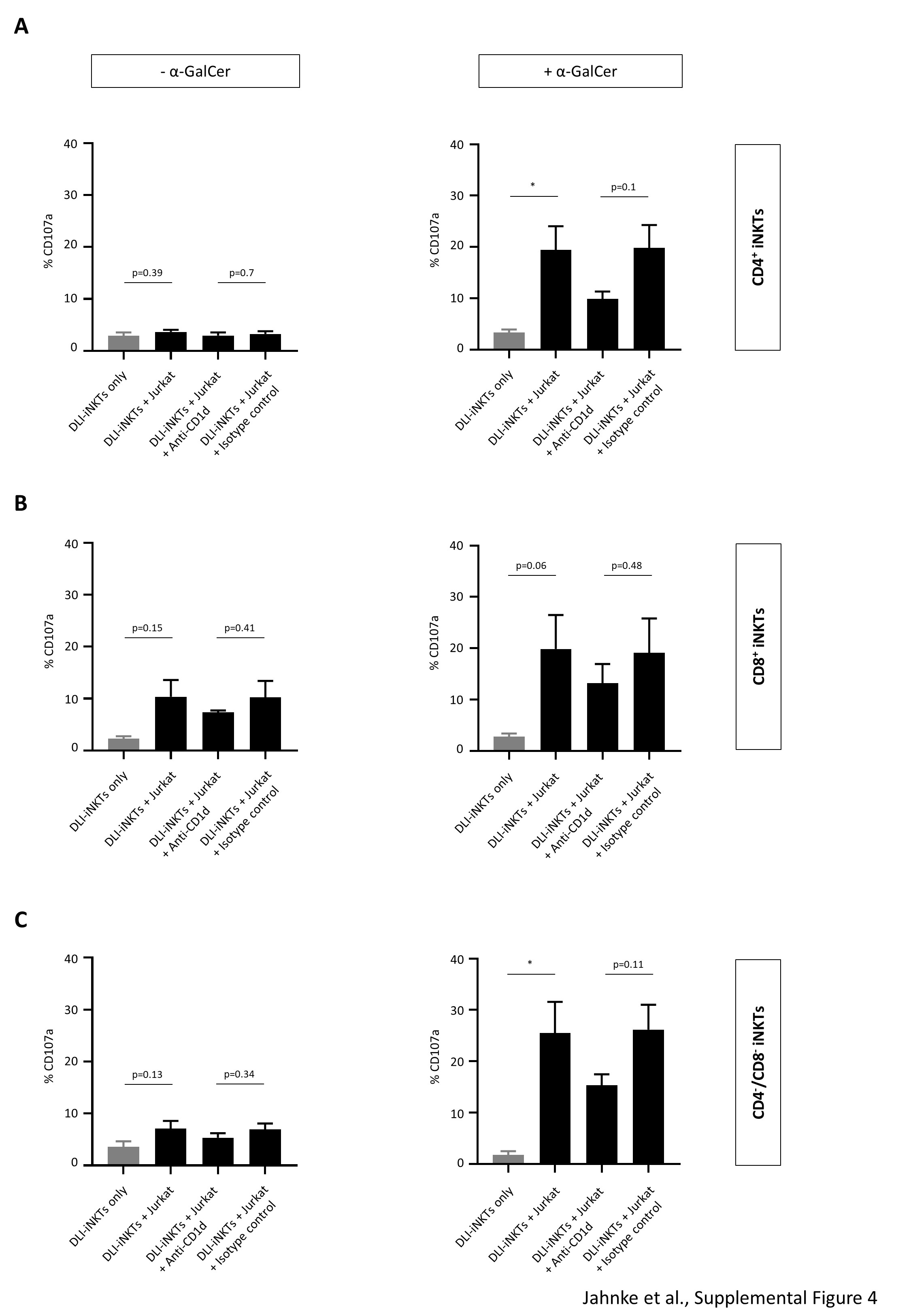

Supplement: Supplemental Figure 4 — Expression of CD107a on DLI-iNKT subsets. Expression of CD107a on (A) CD4+CD8− (B) CD4−CD8+ (C) CD4−CD8− CD3+PBS57-CD1d Tetramer+ DLI-iNKTs after co-culture with Jurkat cells and anti-CD1d or isotype control antibody without and with α-GalCer. For each group n = 3. Bars represent SEM. *p < 0.05. [file Image_4.JPEG]

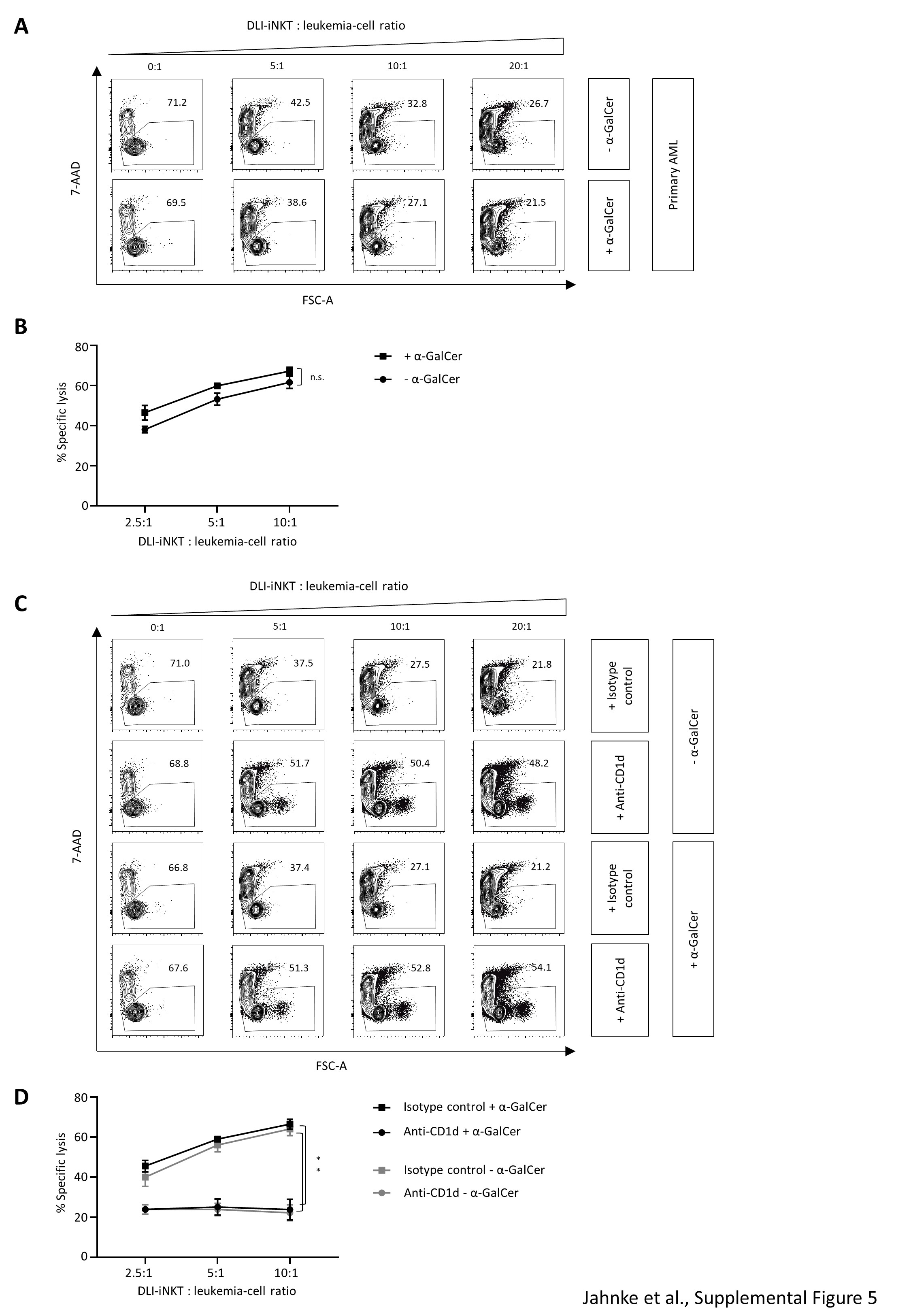

Supplement: Supplemental Figure 5 — Patient AML blasts are lysed by DLI-iNKTs in a CD1d-dependent manner. (A) Representative dot plots and (B) specific lysis illustrating dose-dependent lysis of primary patient AML blasts through culture-expanded DLI-iNKTs in absence and in presence of α-GalCer (n = 3). (C) Representative dot plots and (D) specific lysis of primary patient AML blasts through DLI-iNKTs in presence of anti-CD1d and isotype control antibody together with and without α-GalCer (n = 3). iNKT cells were excluded by gating on PBS57-CD1d Tetramer− cells. Bars represent SEM. *p < 0.05. [file Image_5.JPEG]

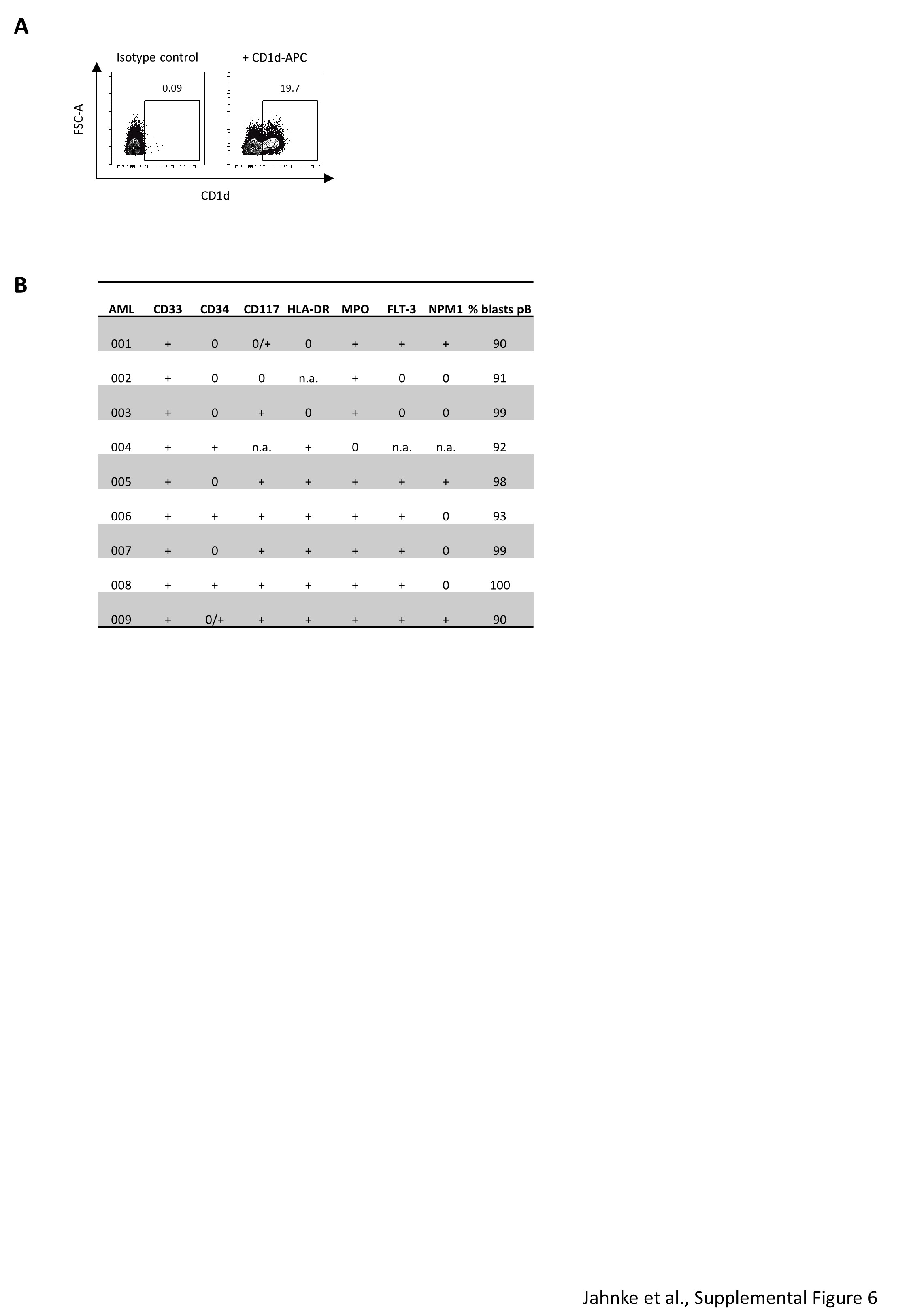

Supplement: Supplemental Figure 6 — Phenotype of patient AML blasts. (A) Representative dot plots of CD1d staining. (B) Immunophenotype of patient AML blasts. 0, negative; 0/+, low; +, positive; n.a., data not available; pB, peripheral blood. [file Image_6.JPEG]
